# Alkaline Phosphatase Profile of Patients with Fibro-Osseous Lesions

**DOI:** 10.22038/IJORL.2022.63776.3185

**Published:** 2022-11

**Authors:** Ekene-Polycarp Onyebuchi, Sunday-Olusegun Ajike, Rasheed Yusuf, Benjamin Fomete

**Affiliations:** 1 *Maxillofacial Surgery Unit, Alex Ekwueme Federal Teaching Hospital, Abakaliki, Ebonyi State, Nigeria.*; 2 *Maxillofacial Surgery Unit, Ahmadu Bello University Teaching Hospital, Zaria, Kaduna State, Nigeria.*; 3 *Chemical Pathology Department, Ahmadu Bello University Teaching, Zaria, Kaduna State, Nigeria.*

**Keywords:** Alkaline phosphatase, Craniofacial, Fibro-osseous, Treatment outcomes

## Abstract

**Introduction:**

Although some studies on craniofacial fibro-osseous lesions have assayed serum alkaline phosphatase levels of affected patients, the findings of these reports are often inconclusive. The aim of this study was to determine the association between the serum ALP levels of individuals with craniofacial fibro-osseous lesions (CFOLs) and treatment outcome.

**Materials and Methods:**

Consecutive patients who presented at the Ahmadu Bello University Teaching Hospital, Zaria from May, 2016 to December, 2017 with lesions histologically diagnosed as CFOLs. The Speight and Carlos’ (2006) classification of CFOLs was adopted, and the serum ALP level of patients and their age- and- gender matched apparently healthy controls were measured at presentation, and repeated at the 3rd and 6th post-operative months for subjects only. Treatment outcomes were assessed 6 months post treatment.

**Results:**

Fifty cases of CFOLs were recorded with a male preponderance, while fibrous dysplasia was the most prevalent lesion, and the maxilla was the most affected jaw (62%). Only 11 subjects had elevated serum ALP levels at presentation, and the mean serum ALP level of subjects with CFOLs was higher (341.2 ± 198.1 IU/L) than that of their age-and gender-matched controls (190.7 ± 110.2 IU/L). With the exception of subjects whose lesions recurred, there was a decrease in the mean serum ALP levels of subjects by the 3rd (245 ± 170.2 IU/L) and 6th (240.5 ± 172.7 IU/L) months post-treatment. Thirty three subjects had elimination of lesions, while three cases each recurred or developed morbidity.

**Conclusion:**

The treatment outcomes of patients with fibrous dysplasia appear to be associated with their serum ALP level. Therefore, serial serum ALP level monitoring suggested in the management of patients with fibrous dysplasia of the craniofacial region.

## Introduction

The fibro-osseous lesions are a group of relatively rare pathological entities which share the same evolutive mechanism characterized by replacement of normal bone by fibrous connective tissue with subsequent deposition of mineralized tissues (1). 

These lesions affect both craniofacial and long bones, although some are found exclusively in the jaws where they may involve single or multiple jaw sites (2). These developmental, neoplastic, reactive and dysplastic pathological processes are subsumed under the rubric of craniofacial fibro-osseous lesions (CFOLs). This includes lesions like fibrous dysplasia, ossifying fibromas and the cemento-osseous (osseous) dysplasias (1). 

However, other lesions with overlapping clinico-pathological and radiological features such as aneurysmal bone cyst, central giant cell granuloma, Cherubism, Brown’s tumour of hyperparathyroidism, chronic sclerosing osteomyelitis and solitary bone cyst have been included by some classification systems (2-4).

Although the etiology of CFOL is largely unknown, mutation in the gene coding for the α-subunit of the GNAS protein located on chromosome 20q13.2-13.3 has been identified in fibrous dysplasia (5), while Pimenta et al. (6) have reported mutations in the HRPT2 gene in lesions of ossifying fibroma. 

Alkaline phosphatase (ALP) is a hydrolase enzyme responsible for dephosphorylation of nucleotides, proteins and alkaloids. It is distributed throughout the body but concentrated in the liver, bile duct, kidney, intestinal mucosa, placenta and bones (7). 

The liver and bone iso-enzymes account for up to 95% of the total serum ALP value (7), and the bone specific ALP isoform is considered a highly specific marker of the bone-forming activity of osteoblasts (7). Normal laboratory value for adults (> 16years) is 64-306 IU/L, while that of children (i.e. ≤ 16 years) is 180-1200 IU/L (7). Markedly elevated serum levels are seen in bone diseases such as Paget’s disease, metastatic bone carcinoma and osteogenic sarcoma (8). 

Other causes of elevated serum ALP include; osteomalacia, physiological growth, biliary obstruction, renal disease, hyperthyroidism, sarcoidosis, haematological malignancies and drugs (NSAIDs, tricyclic antidepressants, oral contraceptive pill, propranolol, methyl Dopa) (8). Although some authors have reported elevated serum levels of ALP in some members of CFOLs (9-11), others did not record changes in the serum ALP levels of individuals with craniofacial fibro-osseous lesions (12-14). 

Therefore, the aim of this study was to determine whether the serum alkaline phosphatase levels of individuals with craniofacial fibro-osseous lesions correlate with the outcome of treatment.

## Materials and Methods 

This was a prospective study conducted on consecutive patients with CFOLs who presented at the Oral and Maxillofacial Surgery clinic of the Ahmadu Bello University Teaching Hospital, Zaria, Kaduna State, Nigeria, between May, 2016 and December, 2017. Biodata, clinical and radiologic findings were reported, and both new and recurrent cases were included in this study. 

Radiological investigations were performed at presentation, and repeated six months post-treatment. 

The definitive diagnosis of each lesion was obtained by juxtaposing the histopathological features with the clinical and radiological features, while the Speight and Carlos’ (2006) classification of fibro-osseous lesions was adopted for tumour classification. Serum ALP level estimation was carried out using the electrophoretic method for both controls and subjects at presentation/pre-operatively, and repeated for only subjects at the 3rd and 6th month after treatment/observation. Individuals who declined surgery were placed on observation while the surgical treatment modalities adopted were: paring down (shaving), excision and jaw resection. Treatment outcomes were determined six months post-operatively/observation using both clinical and radiological measures. Possible outcomes include: tumour elimination, apparent stabilization, recurrence, developed morbidity or mortality. 

Data were analyzed using SPSS version 16 and a p value <0.05 was considered statistically significant at a 95% confidence level, while ethical clearance was obtained from the Health Research and Ethics Committee of the study centre (ABUTHZ / HREC / S17 / 2015).

## Results

There were a total of 10 subjects who were less than 17 years of age and their mean serum 

ALP was higher (599.8 ± 180.1 IU/L) than that of their 10 age-and gender-matched controls (375.4 ± 94.8 IU/L) (Table 1). The differences in the mean serum ALP level of subjects and controls within this age group was found to be statistically significant (p=0.003). Similarly, the mean serum ALP of the 40 subjects whose ages were between 17 and 65 years was higher than that of their age- and gender-matched apparently healthy controls. The difference between the mean serum ALP levels of subjects and controls was statistically significant (P<0.001). 

The overall mean serum ALP of all subjects was found to be higher than that of controls (Table 1), and the difference in the mean serum ALP level of all subjects and controls was found to be statistically significant (P<0.001). 

**Table 1:** Mean serum alkaline phosphatase levels of subjects and controls at presentation

Subjects / controls	n	Serum ALP level (IU/L)		*p*-value
		Mean	S.D	
**Subjects (6 – 16 yrs)**	10	599.8	± 180.1	0.003
**Controls (6 – 16 yrs)**	10	375.4	± 94.8	
**Subjects (17- 65 yrs)**	40	276.6	± 142.7	<0.001
**Controls (17- 65 yrs)**	40	144.5	± 47.5	
**All subjects (6 - 65 yrs)**	50	341.2	± 198.1	<0.001
**All controls (6 – 65 yrs)**	50	190.7	± 110.2	

The serum ALP levels of all controls were within the normal laboratory range at presentation, while 11(22%) of the 50 subjects had elevated serum ALP level. These 11 subjects with elevated serum ALP level consists of 10 (31.3%) cases of fibrous dysplasia (3 recurrent and 7 primary cases), and a single case (5.9%) of ossifying fibroma (Table 2) in a 24-year old female. The mean serum ALP level of these 11 subjects (471.6 ± 127 IU/L) was higher than that of the remaining 39 subjects (304.5 ± 200.1 IU/L) whose serum ALP levels were within the normal laboratory range (P=0.012).

The mean serum ALP of individuals with fibrous dysplasia (402 ± 212.1 IU/L) was higher than that of those with ossifying fibroma (233.4 ± 111.7 IU/L) and osseous dysplasia (231 IU/L) at presentation (Table 2), and the difference in the mean serum ALP levels of members of craniofacial fibro-osseous lesions was statistically significant (P=0.012). Similarly, the mean serum ALP of subjects with Fibrous dysplasia was higher than that of those with ossifying fibroma and osseous dysplasia at the 3^rd^ and 6^th^ months post-treatment (Table 2). 

**Table 2:** Mean serum ALP levels of subjects with fibrous dysplasia, ossifying fibroma and osseous dysplasia at presentation, and at the 3^rd^ and 6^th^ month post-treatment

Tumour type	n	Subjects with elevated serum ALP at presentation n (%)	Mean serum ALP ± SD at presentation (IU/L)	Mean serum ALP ± SD at 3 months (IU/L)	Mean serum ALP ± SD at 6 months (IU/L)
**Fibrous dysplasia**	32	10 (31.3)	402 ± 212.1	297.6±191.2	294.8 ± 194
**Ossifying fibroma**	17	1 (5.9)	233.4 ± 111.7	153.1 ± 50.6	146 ± 43.2
**Osseous dysplasia**	1	0	231	127	109
**Total**	50	11(22)	341.2 ± 198.1	245 ± 170.2	240.5 ± 172.7

The overall mean serum ALP level of the 50 subjects with CFOLs was 341.2 ± 198.1 (IU/L) at presentation, following treatment, there was a reduction in the mean serum ALP level to 245.0 ± 170.2 (IU/L) by the 3rd month, and subsequently to 240.5 ± 172.7 (IU/L) by the 6th month. When analyzed statistically, there was a change in the p-value from 0.265 at presentation, to 0.135 at the 3rd month, and to 0.039 (which is statistically significant) by the 6th month post-treatment. 

Furthermore, there was a steady decline in the mean serum ALP levels of fibrous dysplasia, ossifying fibroma and osseous dysplasia from presentation to the 6th month post-treatment (Table 2). The differences in mean serum ALP levels of members of the CFOL was also found to be statistically significant at the 3rd (p=0.011) and 6th month (p=0.009) after treatment. Similarly, the mean serum ALP value of the (5) subjects who presented with recurrent lesion was higher (400.8 ± 198 IU/L) than that of the remaining 45 subjects with primary lesions (334.6 ± 199.2 IU/L) at presentation. 

Although the mean serum ALP of the cases with primary lesions showed a steady decline through the 3rd (239.7 ± 171.1 IU/L) to the 6th month (232.3 ± 167.4 IU/L) post-treatment, that of those with recurrent lesions had an initial drop at the 3rd month (293.4 ± 173 IU/L), which subsequently became elevated by the 6th month (314.8 ± 221.7 IU/L). 

However, the difference between the mean serum ALP of subjects with primary lesions and those with recurrent lesions was not statistically significant at presentation (P=0.484), at the 3rd (P=0.509) and at the 6th month post-treatment (P=0.316). The mean serum ALP level of the 2 subjects who were under observation (583.5 ± 566.4 IU/L) was higher than that of the remaining 48 cases who had surgical intervention (331.1 ± 177.4 IU/L) at presentation, at the 3rd (566.0 ± 574.2 / 231.7 ± 136.5 IU/L) and the 6th month (541 ± 537.4 / 228 ± 144.7 IU/L). Although there was no statistically significant difference between the mean serum ALP level of those who had surgery and that of those under observation at presentation (P=0.77), there was a statistically significant difference between the mean serum ALP level of subjects who were under observation and that of those who had surgery both at the 3rd month (P=0.005) and 6th month post-treatment (P=0.01).

With respect to treatment outcome and serum ALP level, individuals with recurrent lesions had the highest mean serum ALP level (457.3 ± 234.1 IU/L), followed by those whose lesions were apparently stabilized (305.4 ± 241.6 IU/L) and those that had complete tumour elimination (205.0 ± 127.0 (IU/L) (Table 3).

Retrospectively, subjects with apparent tumour elimination (311.6 ± 165.5 IU/L) were found to have initially presented with the lowest mean serum ALP level, followed by those whose lesions were stable (427.6 ± 269.3 IU/L), while the highest value was reported in the group which had tumour recurrence (443.0 ± 234.6 IU/L). However, while there was a decrease in the mean serum ALP levels of both individuals with tumour elimination or stabilized lesion, that of those with recurrence further increased by the 6th month after treatment. Finally, only 5 subjects (all fibrous dysplasia cases) had elevated serum ALP level at the 6th month post-treatment. This comprises 5.9% of cases who had complete tumour elimination, 18.2% of the apparently stabilized cases, and 66.7% of cases that recurred. There was a statistically significant association between treatment outcome and subjects with elevated serum ALP level at the 6th month post-treatment (P=0.03). 

**Table 3:** Treatment outcomes and mean serum ALP level of subjects at 6 months post-treatment

Outcomes	n	Mean serum ALP (IU/L)	± SD (IU/L)
**Apparently stabilized**	11	305.4	241.6
**Apparently cured**	33	205.0	127.0
**Recurred**	3	457.3	234.1
**Developed morbidity**	3	176.3	72.5
**Total**	50	240.5	172.7

A total of 48 subjects had various surgical treatment modalities, while the remaining 2 cases were placed under observation. These 2 subjects are male patients with histologically diagnosed fibrous dysplasia who were unwilling to undergo any surgical intervention. 

Thirty cases of fibrous dysplasia had surgery, whereas of the 17 individuals with ossifying fibroma, 10 (58.8%) had excision alone, while 6 (35.3%) had jaw resection alone (Table 4). 

On outcomes of treatment, 11 (22%) of the 50 cases of CFOL (all fibrous dysplasias) had apparent stabilization of lesion. This consists of 7 subjects who had shaving, 2 who had excision, and 2 subjects who were under observation. While 33 (66%) of the 50 reported cases of CFOL had elimination of lesions and this comprises 17 fibrous dysplasia cases, 15 ossifying fibroma cases and a case of osseous dysplasia. Three (6%) cases of recurrence were observed (all fibrous dysplasia treated by shaving/paring down), while three (6%) cases developed morbidity.

Although there was a statistically significant association between treatment modality and outcome (P<0.001), the association between diagnosis and outcome was not statistically significant (P=0.072). 

Of the 5 subjects that presented with recurrent lesions, 2 had tumour elimination (subjects who had resection), while 2 (cases that had shaving) were among the cases that recurred and a case that had resection developed morbidity. On the overall, three (30%) of the ten cases of fibrous dysplasia (2 recurrent and 1 primary) who had shaving recurred. 

**Table 4:** Treatment modalities of CFOLs/diagnosis and outcomes

Treatment/diagnosis	Outcomes				
	Apparently stabilized	Tumour Elimination	Recurred	Developed morbidity	Total
**Observation** **Fibrous dysplasia**	2	-	-	-	2
**Shaving** **Fibrous dysplasia**	7	-	3	-	10
**Excision**					
**Fibrous dysplasia**	2	10	-	-	12
**Ossifying fibroma**	-	10	-	-	10
**Osseous dysplasia**	-	1	-	-	1
**Excision + resection** **Ossifying fibroma**	-	-	-	1	1
**Resection alone**					
**Fibrous dysplasia** **Ossifying fibroma**	- -	6 5	- -	1 1	7 6
**Resection + immediate reconstruction** **Fibrous dysplasia**	-	1	-	-	1
**Total**	11	33	3	3	50

## Discussion

*Comparison of Serum Alkaline Phosphatase Levels of Subjects and that of Age and* *Gender- matched Apparently Healthy Controls*

Few studies in the literature have investigated the pattern of serum ALP in individuals with fibro-osseous lesions, however (9, 12-16(, these studies have produced conflicting results. The only case of osseous dysplasia recorded in this study had normal serum ALP level which is in agreement with the reports of More et al., (12) and Gonclaves et al., (16) but the reason for this concordance is not known. However, these studies are case reports (12,16), which are inappropriate for statistical inferences. Moreover, a case of focal osseous dysplasia was presented in this study, unlike the aforementioned studies which were reports on cases of florid osseous dysplasia (12,16).

Most of the subjects with fibrous dysplasia in this study had normal serum ALP level, while elevated serum ALP level was recorded in only 30% of subjects with fibrous dysplasia. The findings of this study partly agrees with the reports of Menon et al., (17) and Delibasi et al. (14) which both found normal serum ALP levels in patients with fibrous dysplasia. The reason for this discrepancy could be partly due to the fewer number of samples reported in the above mentioned reports (14,17). On the other hand, the findings of this study partly agrees with the reports of Regezi (18), Feller et al. (10), and Mahadesh et al. (11) which found occasional elevation of serum ALP in patients with fibrous dysplasia. This could be partly as a result of cases of polyostotic fibrous dysplasia which had elevated serum ALP level as also reported by the aforementioned studies (10,11,18).

Similarly, this study recorded elevated serum ALP level in a single case of ossifying fibroma and this rare finding was also reported in the study of Wang et al. (19) which found elevated serum ALP level in one (a 19-year old female with multi-quadrant lesions) out of the 102 cases of ossifying fibroma reviewed. Although the reason for this finding is unclear, it is interesting to note that both cases occurred in females of child-bearing age. Moreover, MacDonald-Jankowski have stressed that the female sex hormone (20), estrogen stimulates growth of ossifying fibroma lesion and also causes marginal serum ALP elevation. However, while this study presented a case with unilateral mandibular involvement, that of Wang and colleagues had multiple quadrant involvement (19).

Lastly, the mean serum ALP level of subjects with fibrous dysplasia in this study was higher than that of their age-and gender-matched controls, which is in consonance with the report of Cheng et al. (15) which found a higher mean serum ALP level in fibrous dysplasia cases compared to those of their age-and gender-matched controls. The reason for this concordance cannot be ascertained even though both studies compared the serum ALP levels of subjects and that of their age- and gender-matched controls.

**Serum Alkaline Phosphatase Level of Subjects and Tumour Progression**

Generally, there was a decrease in the overall mean serum ALP level from presentation to the 6th month post-treatment. Furthermore, fibrous dysplasia cases who presented with recurrent lesions had a higher mean serum ALP than those with primary lesion. Cases whose lesions were eliminated (all CFOLs) or apparently stabilized (fibrous dysplasia alone) by the 6th month post-treatment had a decrease in their mean serum ALP values at the 3^rd^ and 6^th^ month post-treatment. Conversely, subjects (fibrous dysplasia only) whose lesions recurred had an initial reduction in mean serum ALP level at the 3rd month, which was subsequently elevated six months post-operatively. 

There is paucity of studies in the literature that conducted serial ALP level monitoring, however, the findings of this study are partly in agreement with the report of Cheng et al. (15) which reported a higher mean serum ALP levels in subjects with fibrous dysplasia who presented with recurrent lesion than those with primary lesion. However, the reason for this similarity is unknown. The findings of this study is also in consonance with the report of Park et al. (9) which found elevated serum ALP levels among fibrous dysplasia cases whose lesions recurred following incomplete surgical excision, thus prompting the authors to suggest that serum ALP level may serve as a predictor of progression of fibrous dysplasia (9). This could be partly because both studies conducted serial serum ALP level monitoring unlike most studies where blood samples for serum ALP level determination was only obtained once (11, 17).

Finally, 66.7% of fibrous dysplasia cases that recurred as against only 5.9% of fibrous dysplasia cases with tumour elimination had elevated serum ALP level at the 6th month post-treatment, indicating a possible association between serum ALP level and tumour progression / outcome.

**Outcomes of treatment**

In this study, subjects with fibrous dysplasia whose lesions became apparently stabilized were those that were under observation, and some cases that had shaving/paring down which is partly in agreement with the report of Menon et al. (17) This could be due to the benign nature of this lesion, as well as the short duration of follow-up. On the other hand, subjects with fibrous dysplasia who had resections either had elimination of lesions or developed morbidity (Table 4) which is partly in keeping with the reports of Valentini et al. (21), Park et al. (9), and Cheng et al. (15). The reason for this concordance could be due to complete elimination of lesions (21), which was achieved in this study by placing the resection margins on apparently normal bone with a safety margin. Conversely, subjects whose lesion recurred in this study were recorded among fibrous dysplasia cases that had conservative surgery (Table 4). Although this finding is in support of the reports of Park et al. (14), and Cheng et al. (15), it is at variance with the study of Menon et al. (17) which found stabilization of fibrous dysplasia lesions among individuals who had shaving after a 2-year follow-up period. However, the reason for the above discrepancy is unknown. The 30% recurrence rate observed in this study is also in keeping with the reported rate of 25-50% in the literature (2, 4, 22,23). Although there were two subjects with ossifying fibroma who developed morbidity (Table 4), there was no case of recurrence among the ossifying fibromas in this study which is consistent with the report of Alawi (23), however, this is at variance with the report of Slootweg et al. (24). This could be due to the fact that cases of ossifying fibroma in this study had excisions or resections, unlike that of Slootweg and colleagues (24), where some of the subjects had conservative surgery. As a true neoplasm, ossifying fibroma undergoes continuous growth except if lesions were completely eliminated (25).

There is paucity of data on treatment or outcomes of treatment of the osseous dysplasias probably due to the rare nature of these lesions and in most of the available reports, observation were cited as the main stay of management (23,26). However, some authors (25,27), have stated that complete excision results in no recurrence after 2 years of follow-up, and this is supported by the findings of this study, which had complete excision with no evidence of recurrence as at the last review. Surgical interventions for osseous dysplasias are often complicated by infection (23). Although infection was not observed in this study, its clinical presentation (chronic pus discharge) was sequel to an earlier attempted extraction, which is in agreement with the position of Alawi (23).

Conclusion: The findings of this study have shown that the treatment outcomes of patients with fibrous dysplasia appear to be associated with their serum ALP level. Therefore, serial serum ALP level monitoring is advised in the management of patients with fibrous dysplasia craniofacial region.
